# Fabrication of efficient planar perovskite solar cells using a one-step chemical vapor deposition method

**DOI:** 10.1038/srep14083

**Published:** 2015-09-22

**Authors:** Mohammad Mahdi Tavakoli, Leilei Gu, Yuan Gao, Claas Reckmeier, Jin He, Andrey L. Rogach, Yan Yao, Zhiyong Fan

**Affiliations:** 1 Department of Electronic and Computer Engineering, Hong Kong University of Science and Technology, Clear Water Bay, Kowloon, Hong Kong SAR, China; 2 Department of Physics and Materials Science & Centre for Functional Photonics (CFP), City University of Hong Kong, Hong Kong SAR, China; 3 Shenzhen SOC Key Laboratory, Peking University-HKUST Shenzhen-Hong Kong Institution, Shenzhen 518051, China; 4 Department of Electrical and Computer Engineering, University of Houston, Houston, Texas, 77204, USA

## Abstract

Organometallic trihalide perovskites are promising materials for photovoltaic applications, which have demonstrated a rapid rise in photovoltaic performance in a short period of time. We report a facile one-step method to fabricate planar heterojunction perovskite solar cells by chemical vapor deposition (CVD), with a solar power conversion efficiency of up to 11.1%. We performed a systematic optimization of CVD parameters such as temperature and growth time to obtain high quality films of CH_3_NH_3_PbI_3_ and CH_3_NH_3_PbI_3-x_Cl_x_ perovskite. Scanning electron microscopy and time resolved photoluminescence data showed that the perovskite films have a large grain size of more than 1 micrometer, and carrier life-times of 10 ns and 120 ns for CH_3_NH_3_PbI_3_ and CH_3_NH_3_PbI_3-x_Cl_x_, respectively. This is the first demonstration of a highly efficient perovskite solar cell using one step CVD and there is likely room for significant improvement of device efficiency.

Lead halide perovskite materials, such as CH_3_NH_3_PbI_3_ and CH_3_NH_3_PbI_3-x_Cl_x_, have emerged as attractive candidates for low-cost and efficient solar cells due to their appealing optical and electrical properties^1^,^2^. They can be readily synthesized at low temperature from earth-abundant elements thus greatly lowering the requirement on fabrication facilities^3^,^4^. More importantly, these materials hold promise for high performance photovoltaic devices, i.e. solar cells, due to higher charge carrier mobilities and longer diffusion lengths than many organic semiconductors^5^,^6^. In addition, their band-gap can be conveniently and widely tuned via doping process^7^,^8^,^9^,^10^,^11^. Over the past few years, interest in perovskite photovoltaics has surged, triggered by the fast development of low-cost and efficient lead halide perovskite thin film solar cells^12^,^13^. As a result, the power conversion efficiency (PCE) of this type of solar cells has increased from 3.8% to 19.3% in only 4 years, making them comparable in efficiency to the commercial crystalline silicon solar cells^14^,^15^.

It is known that perovskite materials are of a wide compositional and structural variety which is determined by different metal halide frameworks and the organic constituent species, and this largely influences the properties of perovskite films^7^. Up to now, two general methods, namely, deposition from solution and evaporation from the gas phase have been explored to prepare mesostructured^16^,^17^,^18^,^19^ and planar heterojunction^20^,^21^ perovskite solar cells, respectively. In order to fabricate a perovskite layer by solution methods, three different approaches have been utilized, including one-step deposition of mixed precursors^22^,^23^, sequential solution deposition^24^,^25^ and spray coating^26^. Among evaporation methods, vacuum deposition^27^ and vapor-assisted solution processing^28^ have been used. Among these different fabrication methods, the vacuum co-evaporation of two precursors in one-step is one of the most popular methods to fabricate planar pinholes-free perovskite thin films with good surface coverage and uniformity, which reach a solar cell performance of 12–15% PCE^21^,^28^. However, this technique requires high vacuum, and large scale uniform co-evaporation is also a challenging topic. In this work, we explore the simplified vapor transport approach for perovskite solar cell fabrication, developing a simple one-step chemical vapor deposition (CVD) method to fabricate both triiodide and mixed halide perovskite solar cells with a PCE exceeding 11%. The perovskite layers are synthesized by co-vaporizing two different precursors which are then mixed and transferred to the preheated substrate using Argon as carrier gas in a one-step process to form pinhole-free thin films with excellent surface coverage, a large grain size and long carrier life-time. The CVD approach reported here has great potential for scalable fabrication of perovskite solar cells for practical application in the future.

Figure 1a shows the schematics of the fabrication process of the perovskite thin films employing a CVD tube furnace. Specifically, perovskite thin films were deposited onto a c-TiO_2_-coated FTO glass substrate by a one-step method where lead chloride or lead iodide and methylamine iodide (MAI) were placed in the high temperature zone and the exact position of each of the sources were determined according to their vaporization temperature. In the growth process, the substrates were placed in the left side low temperature zone (Fig. 1a). The perovskites were deposited on the substrates after heating the sources while using Argon carrier gas for both MAI and PbX_2_ vapors with a 70 sccm flow rate. Figures S1a,b show the heating process of source chemicals and the substrate for the fabrication of CH_3_NH_3_PbI_3_ and CH_3_NH_3_PbI_3-x_Cl_x_ perovskite films inside the CVD furnace. In order to improve the quality of the resulting films, optimization of several parameters such as, deposition time, temperature and, annealing process was undertaken. Figure S2 demonstrate the effect of different source temperatures on the quality of CH_3_NH_3_PbI_3-x_Cl_x_ films, with a conclusion that 360 °C is the optimal temperature for our CVD method. To improve the crystallinity of the perovskite materials, an *in-situ* annealing process was performed in the low temperature zone immediately after growth. Figure S3 shows the top view scanning electron microscopy (SEM) images of as-prepared CH_3_NH_3_PbI_3-x_Cl_x_ perovskite films after annealing at different temperatures for one hour. We found that 100 °C (Fig. S3b) is the most suitable annealing temperature which leads to the large grain size of perovskite films with a high crystallinity. At a higher temperature, the perovskite films decompose and in some areas the color changes from brown to yellow, indicating that separate PbI_2_ and perovskite phases coexist in the film.

Figure 2 and S4 display the X-ray diffraction (XRD) and SEM micrographs of CH_3_NH_3_PbI_3-x_Cl_x_ and CH_3_NH_3_PbI_3_ perovskites synthesized by CVD method. Figure 2a compares XRD patterns of FTO, c-TiO_2_, CH_3_NH_3_PbI_3-x_Cl_x_, and CH_3_NH_3_PbI_3_ films. A set of pronounced peaks at 14.08°, 28.41°, 31.85°, and 43.19° are indexed to (110), (220), (310), and (330) planes of an orthorhombic crystal structure of CH_3_NH_3_PbI_3_ perovskite^29^. The peaks at 14.2°, 28.6°, 31.7° and 43.8° are assigned to (110), (220), and (330) planes of CH_3_NH_3_PbI_3-x_Cl_x_ perovskite layer which is in a good agreement with literature^26^. XRD datas indicate a complete reaction of precursors during the CVD process. The morphology of the perovskite thin films is further evaluated by SEM and atomic force microscopy (AFM). As shown in Fig. 2b and S4, the as-synthesized perovskite films on c-TiO_2_-coated FTO substrate have large grain size up to micrometer scale with good surface coverage. The cross-sectional backscattered electron SEM image shows that the films consist of well-defined grains with a thickness of 500 nm (Fig. 2c). The large grain size can be related to volume expansion during the reaction and intercalation of PbX_2_ with MAI, as well as to the rearrangement of the perovskite structure driven by the reduction of grain boundaries due to the minimized grain boundary energy^28^. The root-mean-square (RMS) surface roughness of perovskite films were estimated from AFM images (Fig. 2d) to be 43 nm in the area of 10 μm × 10 μm, which is as small as the roughness of the film fabricated via other evaporation methods regardless of its large grain size. The same set of characterization data for CH_3_NH_3_PbI_3_ perovskite films is presented in Figures S4 and S5. As shown in Fig. S5, energy-dispersive X-ray (EDX) spectroscopy with elemental mapping of carbon, oxygen, nitrogen, titanium, iodine, and lead for the CH_3_NH_3_PbI_3_ perovskite films has a good agreement with literature^28^. In order to study the effect of chlorine in the mixed halide perovskite film, EDX elemental mapping of this atom was performed before and after the annealing process of CH_3_NH_3_PbI_3-x_Cl_x_ film (Fig. S6). The results indicate the existence of Cl at the grain boundaries and the absence of Cl inside the grains after the annealing process. In this case, the initial presence of Cl in the film may greatly influence the crystallization dynamics, favoring a higher order of the organic moieties in the inorganic cage and inducing a preferred orientation of the crystalline grains even in small crystals, in contrast to CH_3_NH_3_PbI_3_. Thus, the presence of Cl helps to minimize the morphological and energetic disorder of the film^30^,^31^,^32^.

Figure 3a,b and S7 show the light harvesting efficiency, photoluminescence (PL), and absorbance spectra of lead halide perovskites prepared by CVD method. The band-gaps for CH_3_NH_3_PbI_3_ and CH_3_NH_3_PbI_3-x_Cl_x_ are estimated to be 1.6 eV (770 nm) and 1.65 eV (750 nm), respectively, which is in a good agreement with previous reports^29^. As both CH_3_NH_3_PbI_3_ and CH_3_NH_3_PbI_3-x_Cl_x_ have broad absorption covering the entire visible range (400–750 nm), they are suitable candidates for photovoltaic applications^33^. Also, it is possible to obtain a high photo-to-electric conversion efficiency due to relatively flat absorption curve in the visible spectrum. As shown in Fig. 3a, the films show only a 10% reflection loss and almost no transmittance in the visible spectral range. Figure 3b shows that CH_3_NH_3_PbI_3_ and CH_3_NH_3_PbI_3-x_Cl_x_ perovskite films emit at 770 nm and 750 nm, respectively. The emission maximum of the mixed halide perovskite is blue-shifted in comparison with the triiodide perovskite material because of its larger bandgap, which is consistent with absorption measurements.

In order to prove that CVD fabricated CH_3_NH_3_PbI_3_ and CH_3_NH_3_PbI_3-x_Cl_x_ perovskite films have desirable electronic properties for solar cell applications, their carrier diffusion length has been estimated from the time resolved PL spectra (Fig. S8), as the efficiency of charge generation and separation in the perovskite films highly depends on the quality of the crystals. By fitting the PL decay curves and using analytical model that was reported elsewhere^5^,^25^ (fitting parameters are presented in Table S1), the PL life-time of CH_3_NH_3_PbI_3_ and CH_3_NH_3_PbI_3-x_Cl_x_ were calculated to be 10 ns and 120 ns, respectively. Due to the long life-time of carriers in the perovskite films, the carriers can easily reach the electrodes in a solar cell device before recombination and therefore increase the power conversion efficiency. Using the diffusion length equation, the carrier diffusion lengths were calculated and are presented in Table 1. CVD fabricated perovskite films have diffusion lengths for both electrons and holes in the range of 100–130 nm and 700–800 nm for CH_3_NH_3_PbI_3_ and CH_3_NH_3_PbI_3-x_Cl_x_ films, respectively which can be attributed to their high crystallinity and good surface coverage^2^,^5^.

We used trihalide and mixed halide perovskite films deposited by CVD on TiO_2_-coated FTO glass for solar cell devices fabrication. The FTO glass was coated with a compact TiO_2_ layer (80–100 nm), followed by deposition of perovskite films by CVD, namely, CH_3_NH_3_PbI_3_ (~300 nm) or CH_3_NH_3_PbI_3-x_Cl_x_ (~500 nm). Thereafter, 2,2′,7,7′-tetrakis(N,N-di-p-methoxyphenylamine)−9,9′- spirobifluorene (Spiro-OMeTAD) was spin coated on the perovskite as a hole transfer layer with a thickness of ~300 nm. Finally, 100 nm of gold was thermally evaporated on Spiro-OMeTAD to complete the device as a back contact electrode. The cross-sectional SEM image of the perovskite solar cell (Fig. 4a) and the schematic band alignment of the constituting layers (Fig. 4b) illustrate the device architecture. Current density-voltage (*J-V*) measurements were performed under simulated AM 1.5 G solar irradiation in air. Figure 4c illustrates the performance of trihalide and mixed halid perovskite solar cells. Based on the *J-V* measurements, CH_3_NH_3_PbI_3-x_Cl_x_ perovskite solar cell fabricated by CVD method showed the highest efficiency of ~11.1% compared with CH_3_NH_3_PbI_3_ perovskite solar cell with a PCE of 9.2%. Note that these *J-V* curves were obtained by the reverse scan and the forward scan results are shown in Fig. S9. It can be seen that there is marginal difference between the forward and the reverse scan. Figure 4d shows the external quantum efficiency (EQE) spectra of the perovskite solar cells. Integrating the overlap of the EQE spectrum with the AM 1.5 G solar photon flux in the range of 300 nm to 900 nm yields maximum current densities of 15.2 mA/cm^2^ and 17.3 mA/cm^2^ for CH_3_NH_3_PbI_3_ and CH_3_NH_3_PbI_3-x_Cl_x_ perovskite cells, respectively. The *J*_*sc*_ results obtained from EQE spectra are slightly lower than those acquired from *J-V* curves (Table 2), possibly due to the single-wavelength illumination of EQE setup compared with the full spectrum solar simulator. The perovskite solar cells were fabricated with different grain sizes and the best efficiency was for perovskite film with an average grain size of ~1 μm. In fact, we have observed that by increasing the grain size up to 5 μm, the PCE started to drop maybe due to the surface recombination^34^. As the thickness of perovskite film is only several hundred nanometers, an average grain size of 1 μm means the material is “single crystalline” in the direction for carrier collection. However, if the grain size is too large, pin holes/gap lines may show up at the grain boundary, this may lead to performance loss.

It is worth noting that the performance of the devices fabricated by CVD method is not comparable to the world record yet. This is primarily due to the fact that we did not fabricate the devices in the glove box because of the constraints of our facility. It is known that perovskite material is highly sensitive to moisture; therefore it is preferable that the entire device fabrication process is performed in glove boxes. Moreover, our device structure is essentially a p-i-n junction structure, unlike the reported devices using mesoporous oxide demonstrating state-of-the-art efficiency^35^. The open circuit voltage of our devices is less than one volt whereas the built-in voltage of the junction in theory should be high (>1 V) due to the band gap of the perovskite (~1.5 eV)^35^,^36^. Here, the low recombination resistance (R_rec_) at the interface of perovskite/TiO_2_ and perovskite/spiro which is voltage (V)-dependent, could be the main reason for low V_oc_. Furthermore, R_rec_ highly depends on fabrication processes of perovskite materials especially one-step method^37^. As a result, the lower R_rec_ at interfaces of perovskite/TiO_2_ and HTM for our one-step deposition, may have higher recombination rate.

In order to optimize the perovskite layer, solar cells with different perovskite film thicknesses have also been fabricated and investigated. As shown in Fig. S10, the optimal absorber thicknesses for CH_3_NH_3_PbI_3_ and CH_3_NH_3_PbI_3-x_Cl_x_ were ~300 nm and ~500 nm, respectively, which was controlled by deposition time during CVD process. This is a natural outcome of the longer carrier diffusion length in CH_3_NH_3_PbI_3-x_Cl_x_ as compared with CH_3_NH_3_PbI_3_. In thin film solar cells, both light absorption and carrier collection efficiencies are crucial factors in determining the final device energy conversion efficiency. The optimal thickness for Cl-free device was 300 nm however the diffusion length of film was 100–130 nm. This is simply because a 130 nm thickness is not enough to absorb sufficient light to generate enough short circuit current, thus a thicker film is required. For Cl-devices, fortunately the diffusion length is 700–800 nm, thus optical absorption is no longer the limiting factor. From carrier collection point of view, a thickness comparable to 700 nm should be good enough, but considering poor transportation and bimolecular recombination^37^,^38^, a 500 nm optimal thickness is also reasonable, which is consistent with the literature^5^.

Moreover, we have found that the surface roughness of FTO substrates has significant effect on perovskite solar cell performance. Figures S11a,b show SEM and AFM images of the commercial FTO substrates, with an RMS surface roughness of 27 nm. In order to reduce the roughness, ion milling was performed on the substrates (see Experimental Section for details), and SEM and AFM images in Fig. S11c,d demonstrate an RMS roughness of only 15 nm after this treatment. Perovskite thin film solar cells using these two kinds of FTO substrates were fabricated by CVD, and their performance comparison can be seen from Fig. S11e. CH_3_NH_3_PbI_3_ based solar cells fabricated on the non-treated FTO substrates demonstrated an efficiency of 6.6%, while the ones fabricated on the ion milled FTO substrate showed an efficiency of 8.2%. The advantage of the smoother FTO substrate may originate from the reduced probability of pinholes formation in the absorber films. Therefore, the aforementioned perovskite devices (Table 2) were all fabricated on the ion milled FTO substrates.

In summary, we have suggested the use of a one-step CVD process to fabricate perovskite materials and heterojunction planar solar cell devices. The perovskite films were deposited onto c-TiO_2_-coated FTO glass via in situ reaction of PbI_2_ or PbCl_2_ and CH_3_NH_3_I vapors. This deposition process produces perovskite layers with large grain size, long carrier diffusion lengths and high surface coverage, which can be used as absorber layers of planar solar cells with a respectable PCE of 11.1%. Overall, our study has shown that the quality of the absorber layer is the key to high efficiency of solar cell devices fabricated by one-step CVD method. The fabrication of thin film perovskites with a large grain size, less grain boundaries, and high uniformity and surface coverage can reduce the recombination of the electrons and holes during their transport through the layer, which leads to a high open voltage. The pinhole free nature of the films results in the suppressed shunting and high fill factor. Since large scale CVD processing is widely used in industry for coating various thin films with high uniformity, our deposition technique should be easily up-scalable, while further improvements of device performance can be achieved by fine tuning composition and band-gap of the mixed halide perovskite film, as well as optimizing the quality of the electron transporting layer.

## Methods

### Methylamine iodide (MAI) preparation

Methylamine iodide (MAI) was synthesized by reaction of 24 mL of methylamine (33 wt% in ethanol, Sigma-Aldrich) and 10 mL of hydroiodic acid (57 wt% in water, Sigma-Aldrich)) in a 250 mL three-neck flask at 0 °C for 2 h. Hydroiodic acid was added dropwise to MAI solution during stirring. The white precipitate was recovered from the solution using a rotary evaporator at 50 °C. MAI powder was dissolved in absolute ethanol and precipitated by adding diethyl ether to the solution. After filtration, the process was repeated several times and finally the white powder was attained and dried at 60 °C in a vacuum oven overnight.

### Device fabrication

FTO glasses with an ohmic sheet resistance of 8 Ω were procured from HARTFORD GLASS, USA. 1 KV argon ion milling at a 75 degree incident angle for 10 minutes was used to reduce the surface roughness of FTO substrates. All substrates were cleaned prior to usage by the following procedure. First, they were immersed in acetone (Merck) and then deionized (DI) water (Milipore, 18 M Ω-cm) containing 3 vol% Triton X-100 and sonicated for 30 minutes for each solution. The specimens were then rinsed with DI water and sonicated in isopropanol for 30 min; rinsed with DI water again, sonicated in a DI water batch for another 30 min and finally dried by an air gas flow. An 80-nm-thick TiO_2_ compact layer was deposited on FTO substrates by spin coating (4000 rpm for 40s) using 0.15 M and 0.3 M solution of titanium diisopropoxide bis(acetylacetonate) (75% in 2-propanol, Sigma-Aldrich) subsequently. After drying at 150 °C for 10 min, they were sintered at 550 °C for 30 min in air, immersed in 50 mM TiCl_4_ (Aldrich) aqueous solutions for 30 min at 70 °C, and rinsed with DI water and ethanol, followed by annealing at 550 °C for 30 min in air to form a compact n-type layer of TiO_2_ (c-TiO_2_).

**Figure 1:**
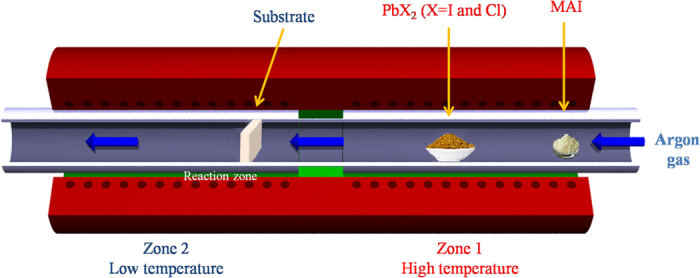
Schematics of the perovskite film fabrication using MAI and PbX_2_ sources deposited onto a c-TiO_2_-coated FTO glass substrate which is performed in a CVD furnace.

In order to synthesize perovskite layer on TiO_2_ substrates, a two inch quartz tube furnace (KJMTI OTF1200X) in a vacuum with an inert carrier gas (Argon) which had two separate zones was used. Subsequently, the perovskite materials were fabricated through dual-source evaporation from lead chloride (PbCl_2_) or lead ioddide (PbI_2_) and methylammonium iodide (CH_3_NH_3_I) simultaneously, onto the TiO_2_-compact-layer-coated FTO substrates using Argon carrier gas, followed by the heating of the substrates to complete the reaction. Custom made quartz crucibles were used for CH_3_NH_3_I and PbX_2_ (X = I and Cl) powders inside the tube furnace to confine the vapor. The vacuum and Ar flow rate were around 1 mTorr and 70 sccm, respectively. 500 mg of CH_3_NH_3_I and 100 mg of PbX_2_ (X = I and Cl) were put into separate crucibles in the upper flow right zone of the furnace (Fig. 1a). We found that the suitable positions of the CH_3_NH_3_I and PbX_2_ sources are at a 19 cm and 9 cm distance from the right side of the middle thermal isolator according to their melting point temperature. The TiO_2_-compact-layer-coated FTO substrates were placed in the down flow left zone at 80 °C. The temperature of the two sources was gradually ramped up to 120 °C, 300 °C or 360 °C for CH_3_NH_3_I, PbI_2_ or PbCl_2_, respectively. Perovskite layers were optimized for best device efficiency by varying different key parameters such as the gas flow rate, temperature, time and the amount of two different sources. After fabrication of perovskite layer, the *in-situ* annealing process was performed in the second zone of the furnace immediately after deposition at 100°C for 60 minutes.

Spiro-OMeTAD (Lumtec, Taiwan) was used as the hole-transporter layer of the solar cells. It was deposited by a spin coating process (3,000 r.p.m. for 40 s) using spiro/chlorobenzene (80 mg/1 mlit) with 17.5 μl Li-bis(trifluoromethanesulfonyl) imide (Li-TFSI)/acetonitrile (500 mg/1 ml) and 28.5 μl TBP as additives. Before completing the fabrication process, the devices were left in a desiccator overnight and finally, a gold counter electrode was deposited by thermal evaporation (0.08 nm/s). The unsealed devices were tested in air immediately after the cathode deposition.

### Film characterization

The thickness, morphology and roughness of the films were studied by field-emission scanning electron microscopy (FESEM, Hitachi S4160, Japan) equipped with energy-dispersive X-ray spectroscopy (EDS). Phase characterization was performed by X-ray diffraction method (Bruker D8 X-ray Diffractometer, USA) utilizing a Cu *Kα* radiation. The optical absorption was recorded on a Varian Carry 500 spectrometer (Varian, USA) and a home-made UV-visible setup using an integrating sphere with a broadband halogen light source. The reflectance + transmittance (R + T) were obtained by putting the sample inside the integrating sphere, and only transmittance (T) was measured by putting the sample right at the entrance for the light beam on the integrating sphere. Afterwards, the absorption (A) spectra was achieved by subtracting reflectance and transmittance from unity using the following equation: A = 1−(R + T).

AFM measurements of the sample surfaces were acquired using a Veeco (Santa Barbara) Dimension 3100, operating in tapping mode. Steady-state photoluminescence spectra and time-resolved photoluminescence decays were measured on an Edinburgh Instruments FLS920P fluorescence spectrometer. A picosecond pulsed diode laser (EPL-670, 671.4nm excitation wavelength, pulse width 62.8ps) was used as excitation source and a long pass filter with 730nm cut-on wavelength was put in the emission path to eliminate stray light hitting the R928T detector. The excitation laser beam was focused on the reacted area of the solid sample. Photoluminescence decay curves were collected using time-correlated single photon counting. Lifetimes were extracted by tail-fitting a three-component exponential function of the 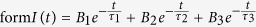 to the decay curves, where 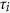 is the 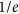 lifetime constant and 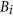 the amplitude for each exponential component. Amplitude weighted average lifetimes were calculated for each fit by taking the contraharmonic mean of each component: 
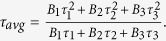


### Device characterization

The solar spectrum at AM1.5G was simulated by an Abet Class AAB Sun 2000 simulator with an intensity of 100 mWcm^−2^ calibrated with a KG5-filtered Si reference cell. The current density-voltage (J-V) data was measured using a 2400 Series SourceMeter (Keithley, USA) instrument. J-V sweeps were performed between 0 and + 1.2 V, with a step size of 0.02 V and a delay time of 150 ms at each point. External quantum efficiency (EQE) spectra were recorded versus wavelength under a constant white light bias of nearly 5 mW.cm^−2^ using Oriel QE-PV-SI (Newport Corporation).

## Additional Information

**How to cite this article**: Tavakoli, M. M. *et al.* Fabrication of efficient planar perovskite solar cells using a one-step chemical vapor deposition method. *Sci. Rep.* **5**, 14083; doi: 10.1038/srep14083 (2015).

## Supplementary Material

Supplementary Information

## Figures and Tables

**Figure 2:**
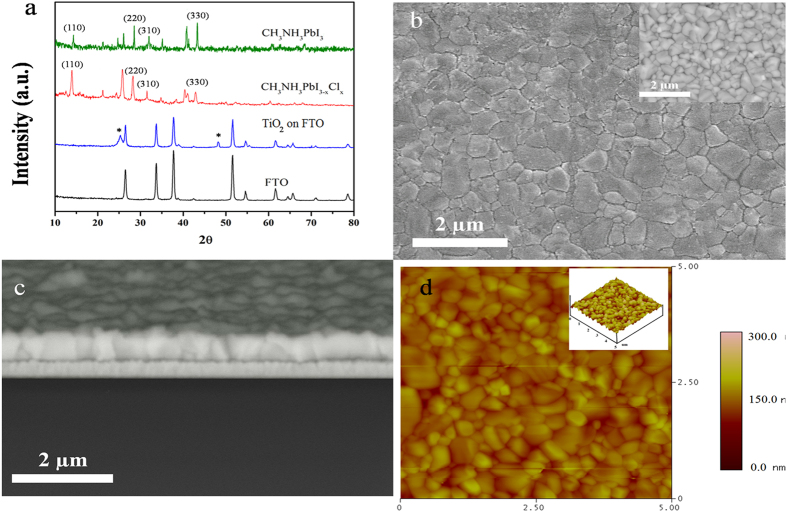
Structural characterization of the perovskite films deposited on c-TiO_2_-coated FTO substrates using CVD method: (**a**) XRD patterns of FTO, TiO_2_ film on FTO substrate (the peaks labeled with “*” are from TiO_2_), CH_3_NH_3_PbI_3-x_Cl_x_ and CH_3_NH_3_PbI_3_; (**b**) top-view secondary electron SEM image of a CH_3_NH_3_PbI_3-x_Cl_x_ layer, with an inset showing backscattered electron (BSE) image with higher resolution; (**c**) cross-sectional BSE SEM image of a CH_3_NH_3_PbI_3-x_Cl_x_ layer; (**d**) AFM height images (10 × 10 μm) with an inset showing 3D topographic image.

**Figure 3:**
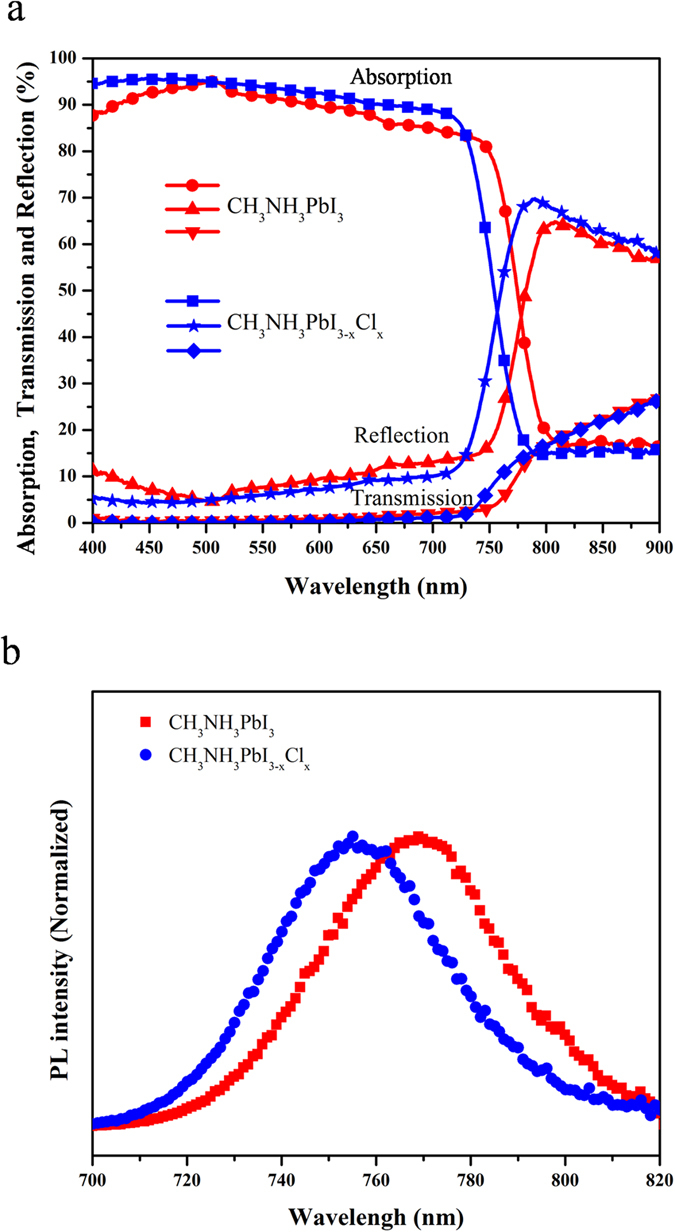
Optical (**a**) and photoluminescence (**b**) spectra of lead halide perovskite films.

**Figure 4:**
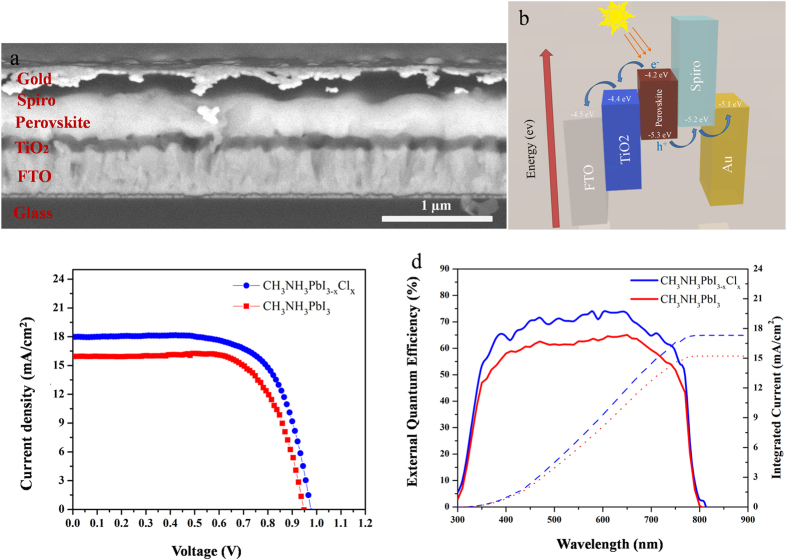
(**a**) Cross-sectional SEM micrograph of a CVD deposited perovskite solar cell with annotated layer structure. (**b**) Schematics of energy levels of the materials constituting the solar cell. (**c**) Current density−voltage (*J-V*) characteristics of the solar cell devices under AM 1.5G illumination, (**d**) EQE curves (solid line) and the integrated photocurrent (dash line) of CH_3_NH_3_PbI_3_ and CH_3_NH_3_PbI_3-x_Cl_x_ perovskite solar cells under AM 1.5G irradiation.

**Table 1:** Carrier life-time and diffusion length of the perovskite films prepared by CVD which were derived from the time-resolved PL data using the model of Ref. 5.

**Perovskite**	**Carrier**	**D(cm^2^/s)**	**τ_s_(ns)**	**L_D_(nm)**
CH_3_NH_3_PbI_3-x_Cl_x_	Electrons	0.042 [5]	120	709
	Holes	0.054 [5]		804
CH_3_NH_3_PbI_3_	Electrons	0.017 [5]	10	130
	Holes	0.011 [5]		105

**Table 2:** Performance of solar cell devices based on CH_3_NH_3_PbI_3-x_Cl_x_ and CH_3_NH_3_PbI_3_ perovskite materials synthesized by one-step CVD method under simulated AM 1.5 G irradiation.

**Perovskite**	**V_oc_ (V)**	**Jsc (mA/cm^2^)**	Jsc (mA/cm^2^)from EQE	**Fill Factor**	**PCE (%)**
CH_3_NH_3_PbI_3-x_Cl_x_	0.97	18	17.3	0.64	11.1
CH_3_NH_3_PbI_3_	0.95	15.9	15.2	0.61	9.2
