# Supplementary material for: Fabrication of efficient planar perovskite solar cells using a one-step chemical vapor deposition method
Source: Sci Rep. 2015 Sep 22;5:14083. doi: 10.1038/srep14083 (PMC4585726; doi:10.1038/srep14083)
Supplement: Supplementary Information [file srep14083-s1.doc]

**Supplementary Information for:**

**Fabrication of efficient planar perovskite solar cells using a one-step chemical vapor deposition method**

Mohammad Mahdi Tavakoli^1^, Leilei Gu^1^, Yuan Gao^1^, Claas Reckmeier^2^, Jin He^3^, Andrey L. Rogach^2^, Yan Yao^4^ and Zhiyong Fan^1^*

*^1^Department of Electronic and Computer Engineering, Hong Kong University of Science and Technology, Clear Water Bay, Kowloon, Hong Kong SAR, China*

*^2^Department of Physics and Materials Science & Centre for Functional Photonics (CFP), City University of Hong Kong, Hong Kong SAR, China*

*^3^Shenzhen SOC Key Laboratory, Peking University-HKUST Shenzhen-Hong Kong Institution, Shenzhen 518051, China*

*^4^Department of Electrical and Computer Engineering, University of Houston, Houston, Texas, 77204, USA*

*Corresponding author, email: [eezfan@ust.hk](mailto:eezfan@ust.hk) (Z. Fan).


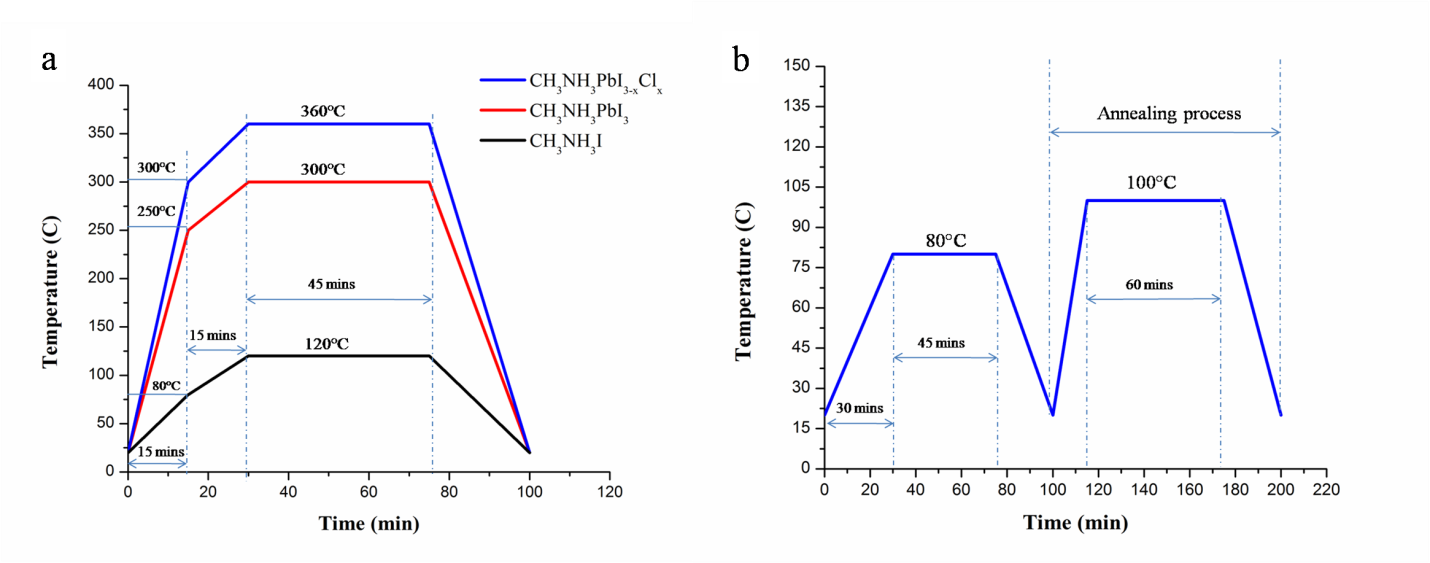


**Figure S1.** The heating process of source chemicals (a) and the substrate (b) for the fabrication of CH_3_NH_3_PbI_3_ and CH_3_NH_3_PbI_3-x_Cl_x_ perovskite films inside the CVD furnace

**
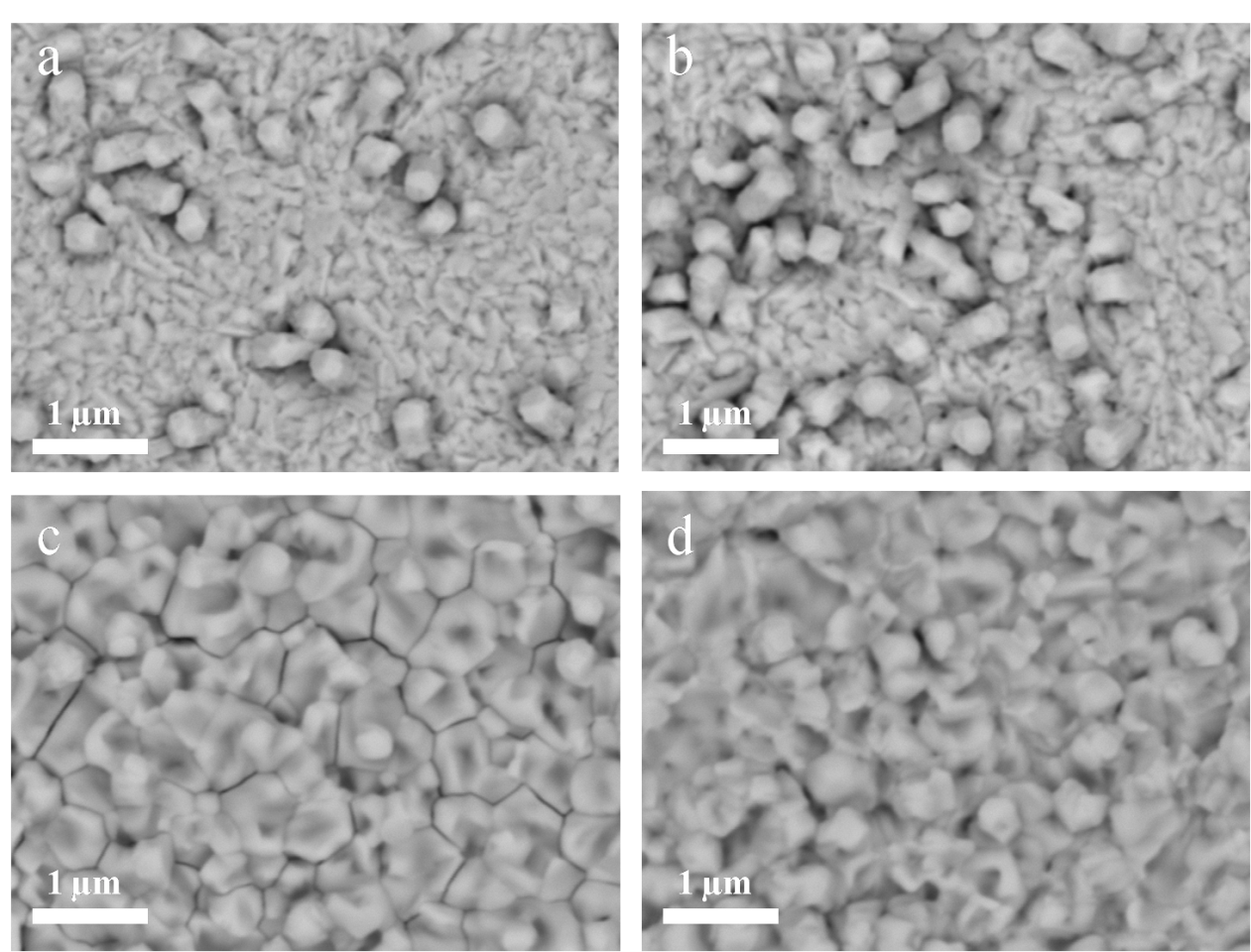
**

**Figure S2.** Top-view BSE SEM images of CH_3_NH_3_PbI_3-x_Cl_x_ films fabricated by CVD method at different temperatures ; (a) 250°C (b) 300°C (c) 360°C (d) 400°C.


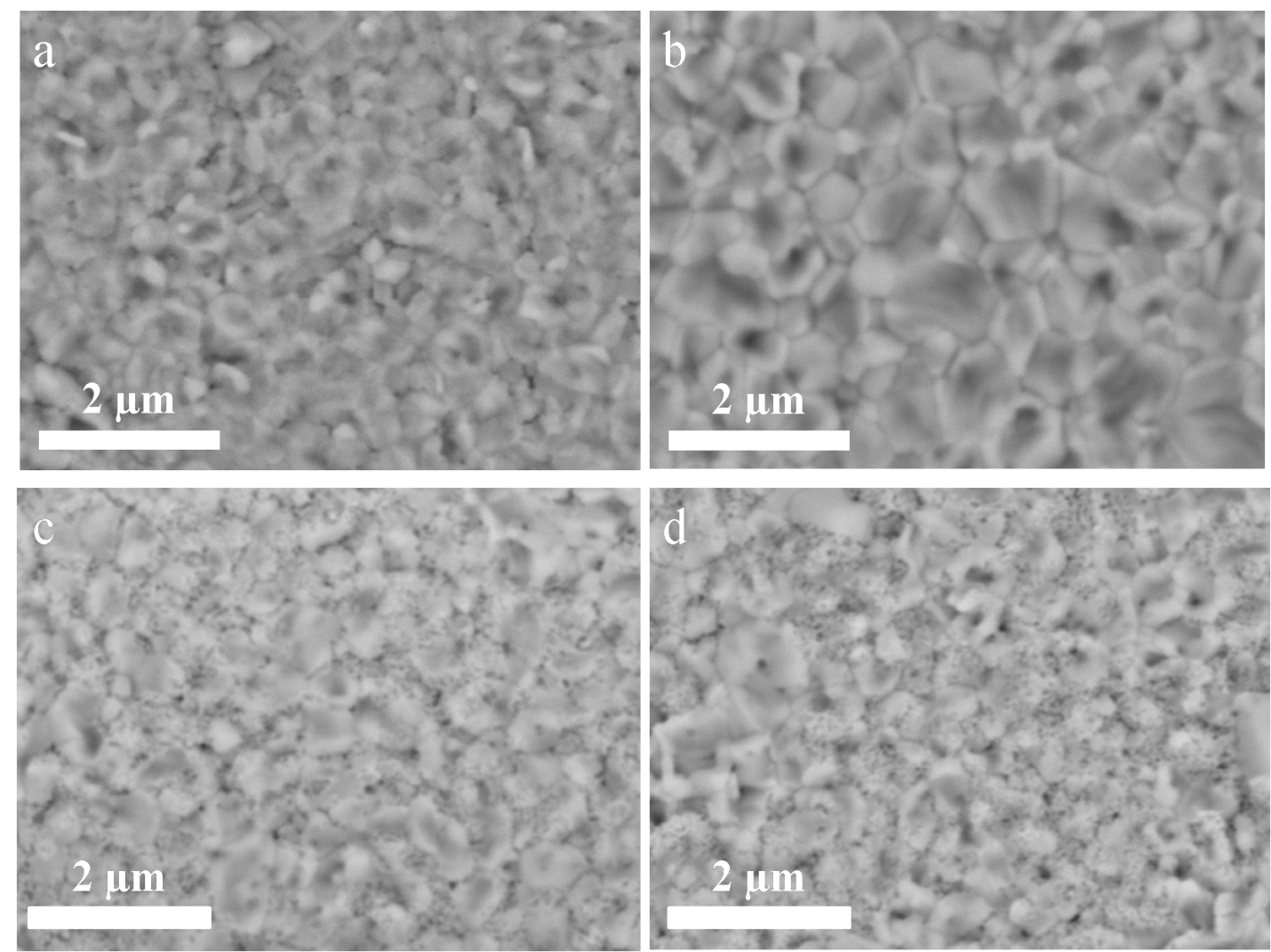


**Figure S3.** Top-view BSE SEM images of CH_3_NH_3_PbI_3-x_Cl_x_ films after annealing process using different temperatures for one hour; (a) 80°C (b) 100°C (c) 130°C (d)150°C.


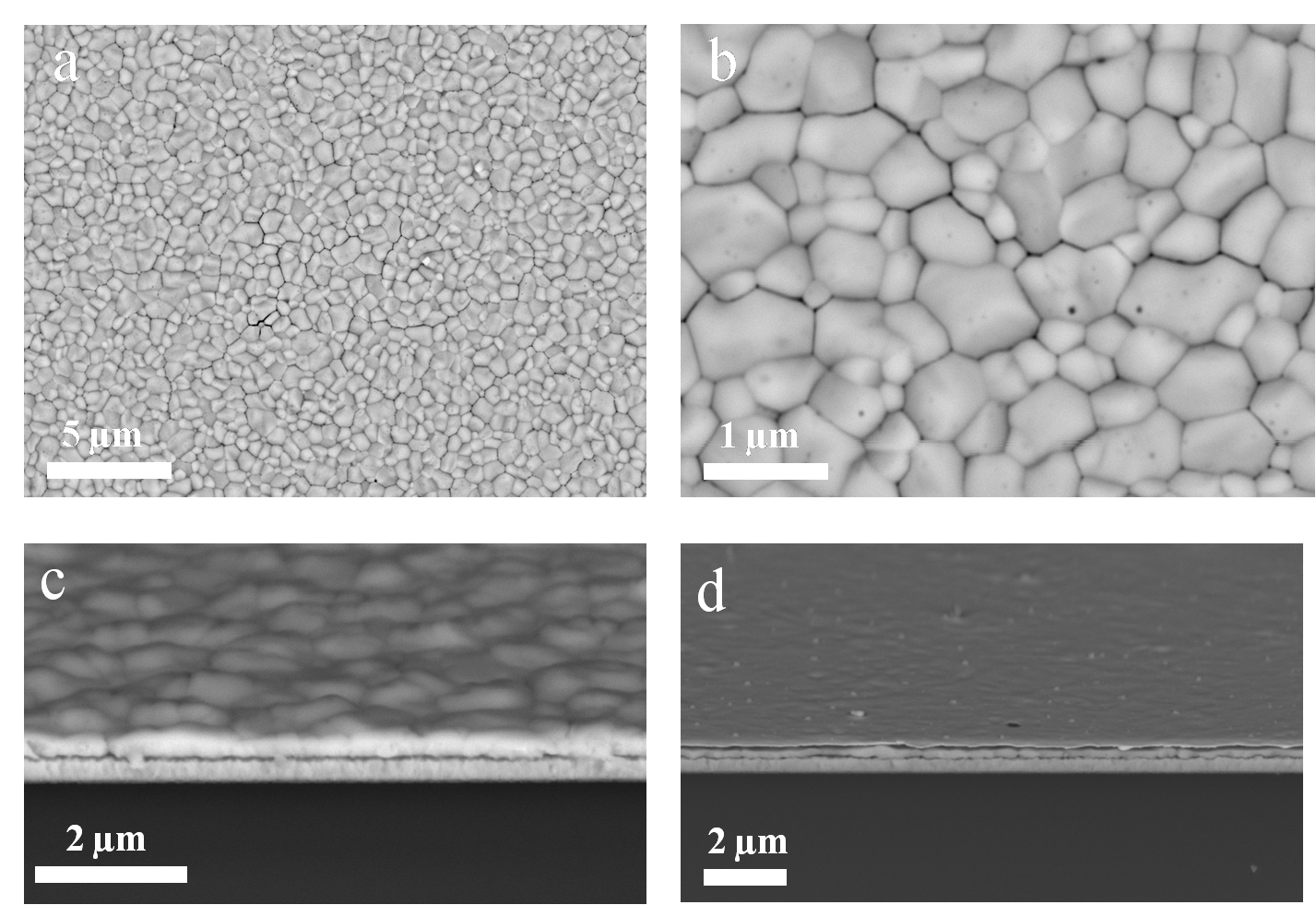


**Figure S4.** Perovskite thin film deposited on the c-TiO_2_-coated FTO substrate using CVD method: top-view BSE SEM images of CH_3_NH_3_PbI_3_ layer, (a) low magnification (b) high magnification; cross-sectional BSE SEM images of (c) a CH_3_NH_3_PbI_3_ thin film and (d) a CH_3_NH_3_PbI_3_ perovskite solar cell.


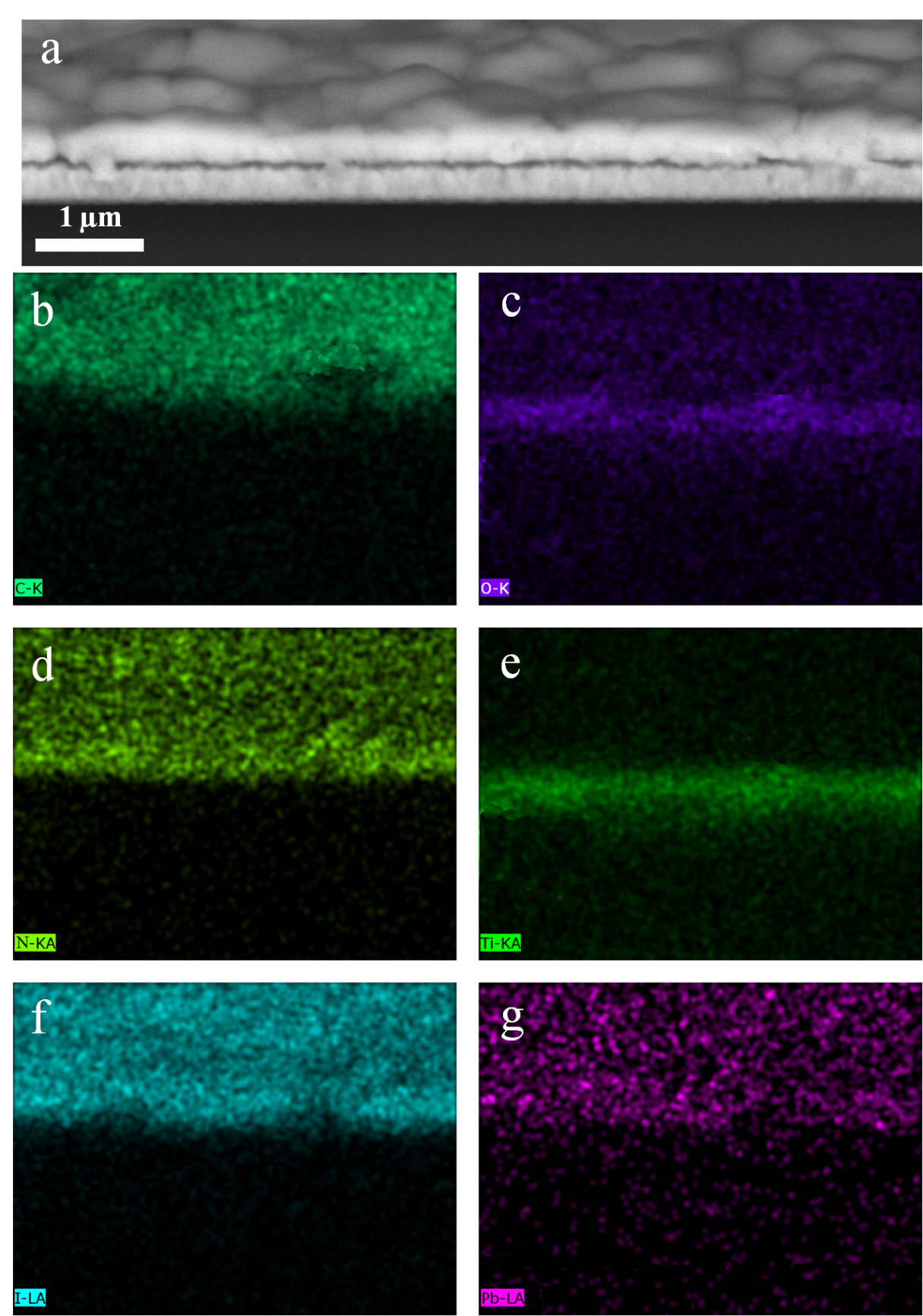


**Figure S5.** Cross-sectional BSE SEM image (a) and energy-dispersive x-ray (EDX) spectroscopy with elemental mapping of carbon (b), oxygen (c), nitrogen (d), titanium (e), iodine (f), and lead (g) for a CH_3_NH_3_PbI_3_ perovskite film.


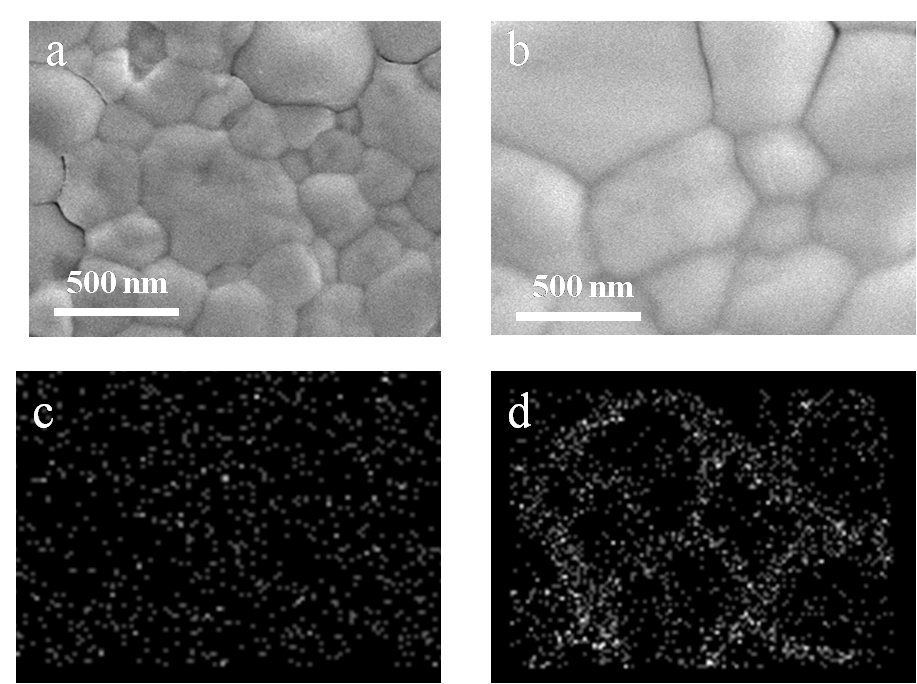


**Figure S6.** Top view SEM images of a CH_3_NH_3_PbI_3-x_Cl_x_ perovskite film and its energy-dispersive x-ray (EDX) spectroscopy with elemental mapping of chlorine (a), (c) before, and (b), (d) after the annealing process.


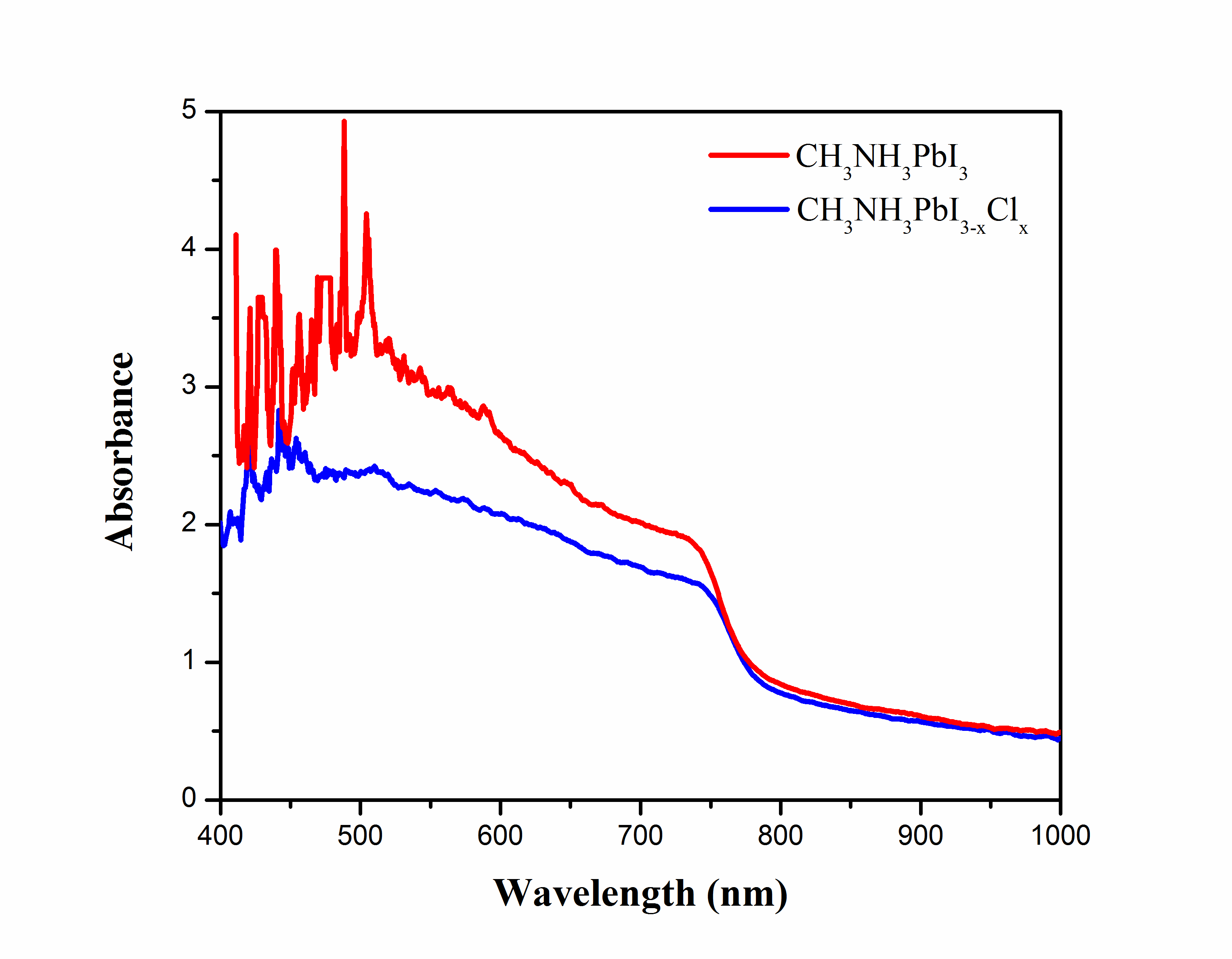


**Figure S7.** The absorbance spectra of perovskite films: CH_3_NH_3_PbI_3_ (red curve) and CH_3_NH_3_PbI_3-x_Cl_x_ (blue curve).


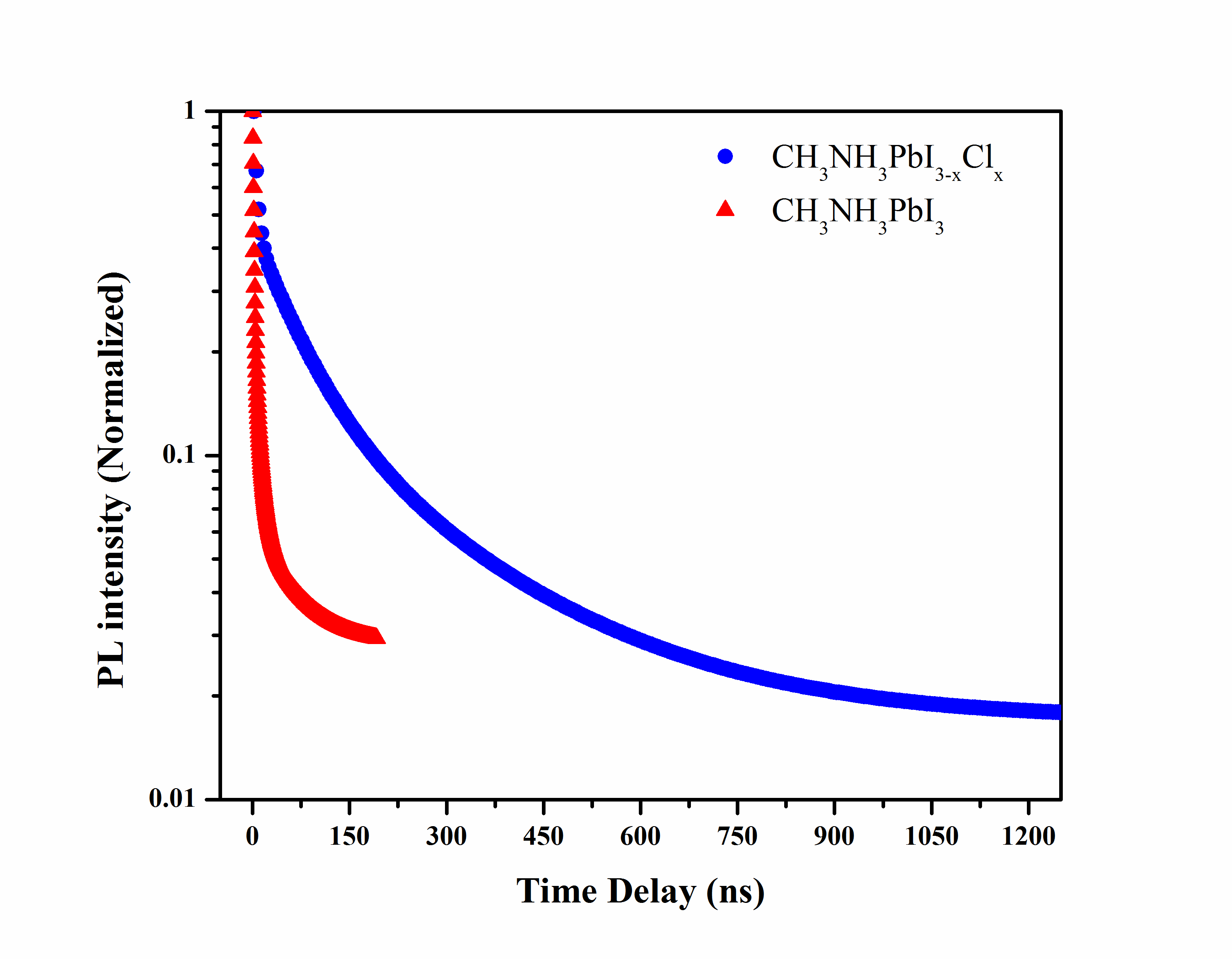


**Figure S8.** Time-resolved PL decays (excitation wavelength 670 nm) of CH_3_NH_3_PbI_3_ and CH_3_NH_3_PbI_3-x_Cl_x_ perovskite films synthesized by CVD.

**Table S1.** Fitting parameters of the corresponding PL decay curves

| **Sample** | ***τ* (ns)** | ***β_1_*** | ***Β_2_*** | ***Β_3_*** | **χ²** |
| --- | --- | --- | --- | --- | --- |
| CH_3_NH_3_PbI_3-x_Cl_x_ | 120 | 16872.6895 | 6148.4697 | 2820.6443 | 1.396 |
| CH_3_NH_3_PbI_3_ | 10 | 17727.48 | 4068.8584 | 829.4182 | 1.445 |


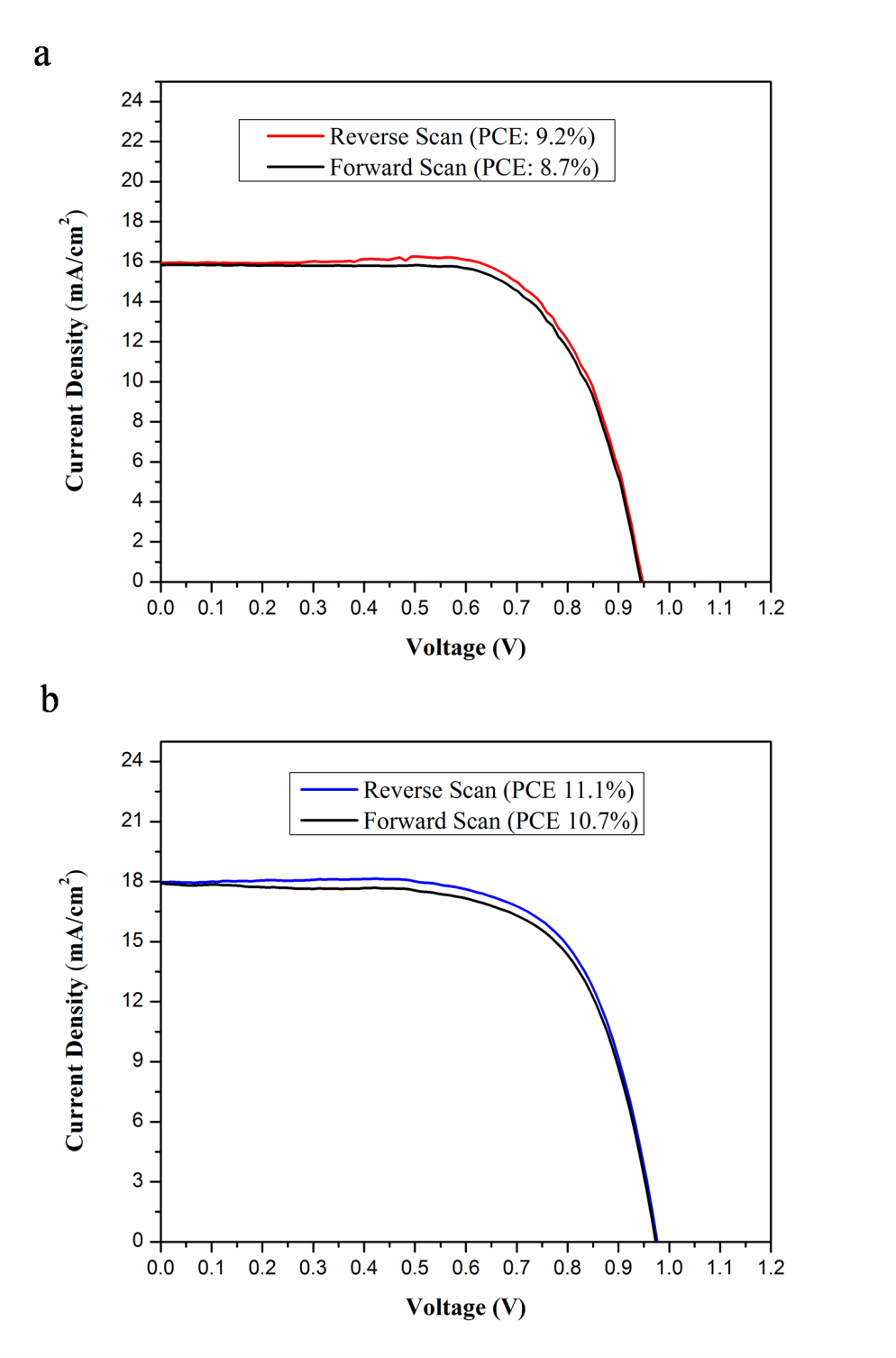


**Figure S9.** Current-density versus voltage characteristics for perovskite solar cells (a) CH_3_NH_3_PbI_3_ and (b) CH_3_NH_3_PbI_3-x_Cl_x_ measured under different scan directions. Efficiency values are shown in each figure legend.

**
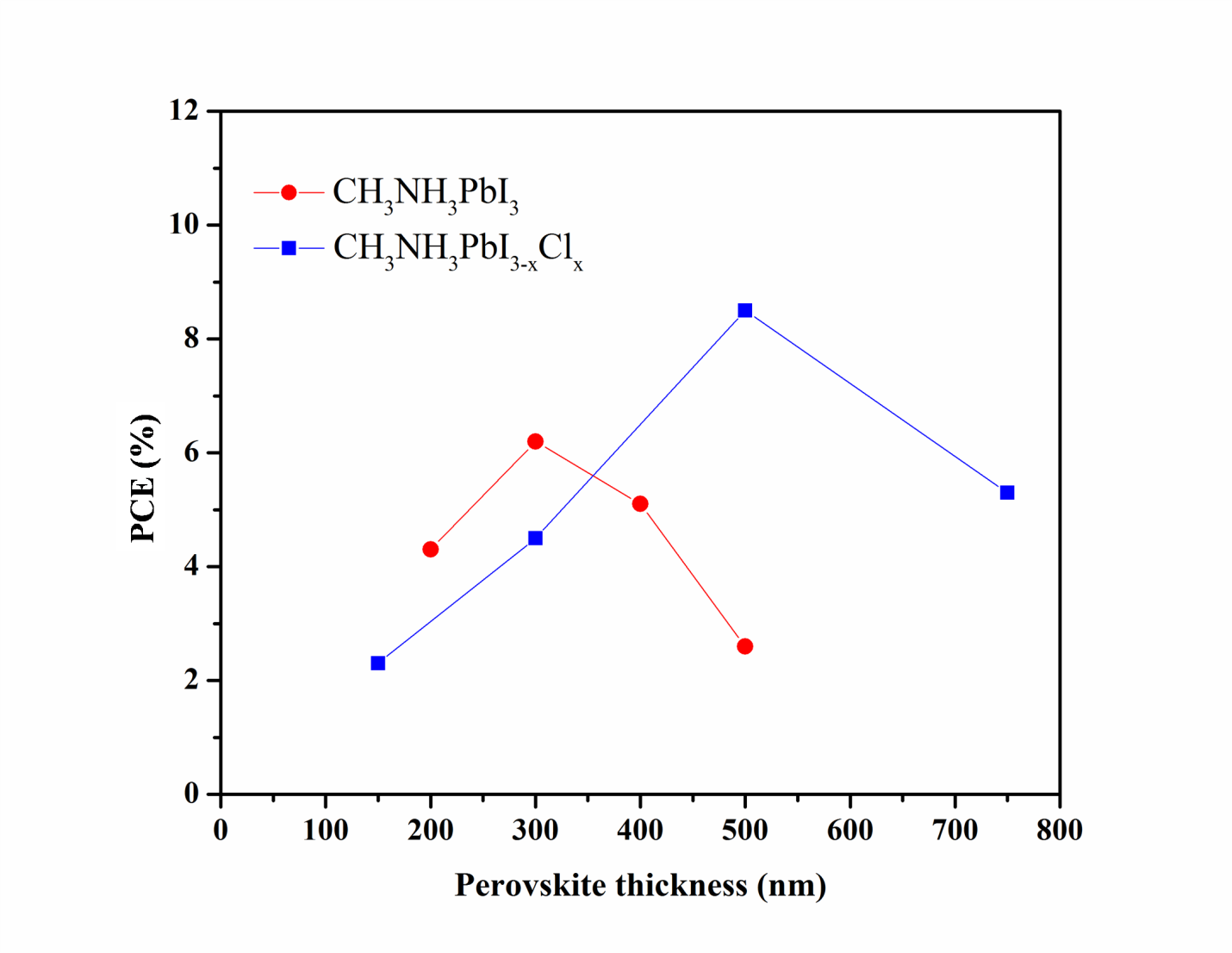
**

**Figure S10.** The effect of the layer thickness of trihalide and mixed halide perovskite films on the efficiency of solar cell devices.

**
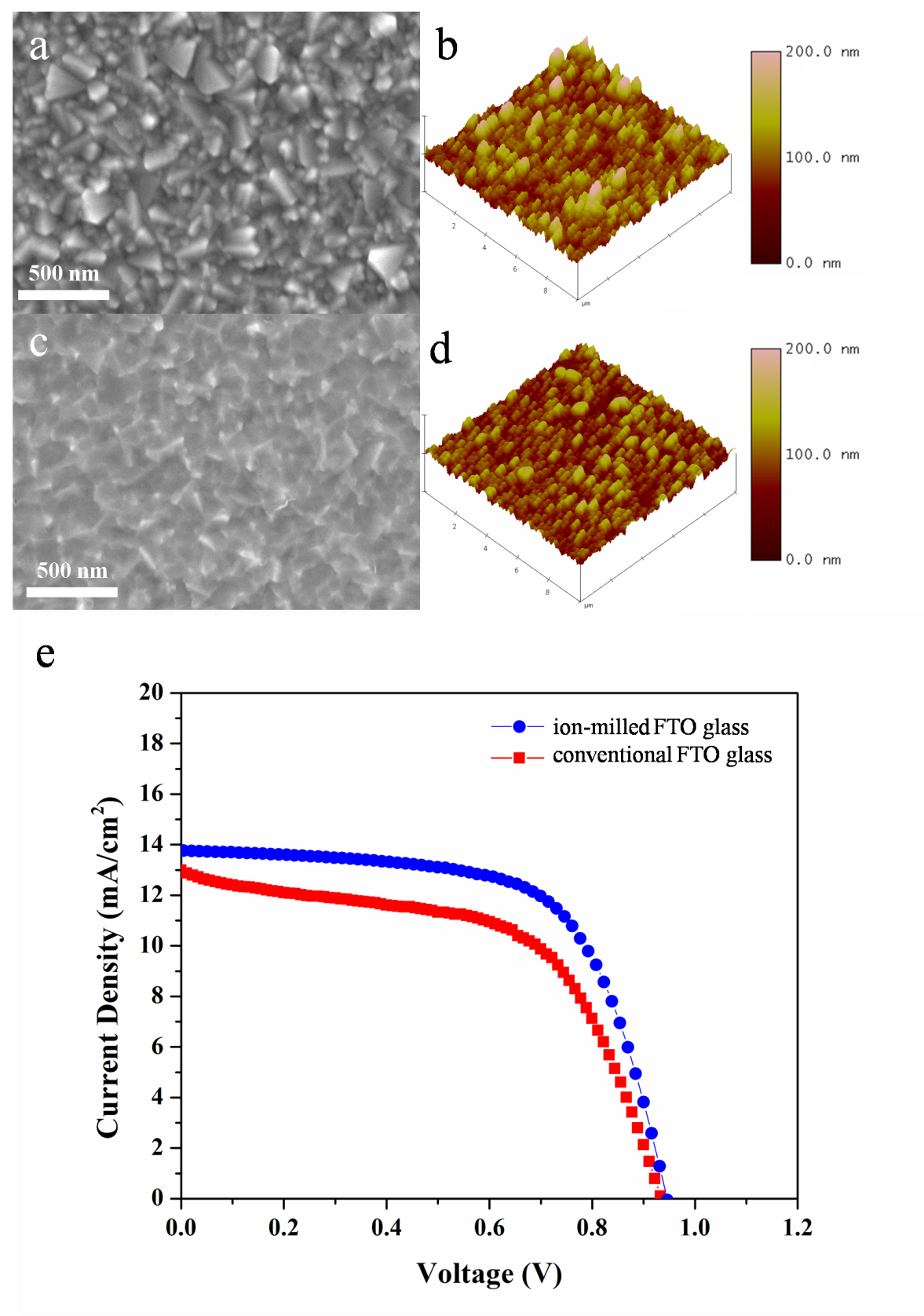
**

**Figure S11.** The effect of ion milling process of FTO glasses on the J-V characteristics and the surface roughness of device; top view SEM images and 3D topographic AFM images of (a,b) conventional FTO glass and (c,d) ion milled FTO glass; (e) J-V measurement of as-synthesized CH_3_NH_3_PbI_3_ perovskite film on conventional FTO glass (red curve) and ion-milled FTO glasses (blue curve).
